# Editorial: Bacterial Secretion Systems, Volume II

**DOI:** 10.3389/fmicb.2022.917591

**Published:** 2022-05-24

**Authors:** Ignacio Arechaga, Eric Cascales

**Affiliations:** ^1^Departamento de Biología Molecular, Instituto de Biomedicina y Biotecnología de Cantabria (IBBTEC), Universidad de Cantabria-CSIC, Santander, Spain; ^2^Laboratoire d'Ingénierie des Systèmes Macromoléculaires, Institut de Microbiologie, Bioénergies et Biotechnologie, CNRS—Aix-Marseille Université, Marseille, France

**Keywords:** secretion systems, bacteria, structural biology, microbiology, biochemistry

In 2019, a first volume of the Research Topic “*Bacterial Secretion Systems*” was published in Frontiers in Microbiology. This volume was comprised of 22 articles covering different aspects of bacterial secretion systems, offering a broad view on these multiprotein complexes responsible for the delivery of effectors in the environment or into target cells. In this second issue, which groups 15 articles, further insights on these fascinating nanomachines are uncovered.

Bacteria have developed sophisticated machineries to interact with the surrounding environment and with other cells. Some of these systems, like the Tat export pathway or the Sec machinery, broadly distributed in bacteria, are dedicated to protein translocation across or protein insertion into the cytoplasmic membrane. In addition, bacteria have evolved a vast repertoire of secretion pathways to transport effectors across the cell envelope. Up to date, 11 different secretion pathways (T1SS–T11SS), involved in substrate transport, protein exposition at the cell surface, pili assembly and motility, have been described. The T1SS, T3SS, T4SS and T6SS are large multiprotein complexes that span both the inner and outer membranes of Gram-negative bacteria and are thus capable to translocate effectors in a single step. Other systems, such as T2SS, T5SS, T9SS and T11SS require the assistance of the Sec or Tat export pathway to export first effectors in the periplasm before being selected by the secretion apparatus. Bacterial secretions systems can deliver a broad range of effectors, from small molecules to large macromolecules such as proteins and DNA. The understanding of the biogenesis and the mechanism of action of these secretion systems requires a combination of microbiology, genetics, biochemistry and biophysical approaches. This issue is constituted of four reviews and 11 research articles including two comprehensive bioinformatics studies.

Troman and Collinson provide an extensive review describing the journey of a protein across the cell envelope, emphasizing the molecular complexes responsible for protein export and insertion in the inner and outer membranes, the role of general or dedicated chaperones that assist protein folding and perform quality control and eventually degradation, and how the energy of the inner membrane is mobilized for outer membrane processes.

Two research articles provide novel information on protein export and inner membrane insertion pathways. Zhao et al. report the characterization of a suppressive mutation isolated when the signal recognition particle responsible for co-translational export is missing. They found that this mutation localizes in the Shine Dalgarno sequence of a gene encoding the S10 ribosomal protein, hence leading to a decrease in protein translation rate and accuracy. Mishra and Brady focus on the two YidC proteins encoded by *Streptococcus mutans*, YidC1 and YidC2. By targeting the less-conserved cytoplasmic domains of these proteins and engineering YidC1/YidC2 chimeras, they define specific contributions of these two paralogs in various growth conditions.

Then, Mekasha and Linke provide a comprehensive review on secretion systems present in bacterial fish pathogens. They show that, such as their plant- or animal-pathogen counterparts, these pathogens use classical secretion systems, to deliver effectors that are key to the infection process. They note an overrepresentation of T6SS and T9SS in Bacteroidetes fish pathogens and of T1SS, T2SS, T3SS and autotransporter pathways in Proteobacterial fish pathogens.

Otten et al. provide novel information on the under characterized platform of plant pathogen T3SSs. They identified an internal translation start site in the *hrcQ* gene, leading to the synthesis of a shorter, C-terminal protein, HrcQ_C_. This C-terminal domain interacts with and stabilizes the full-length HrcQ protein, and colocalizes with it at the T3SS. Finally, interactions between HrcQ_C_ and the HrcD and HrpB4 T3SS components were found, suggesting that in addition to its chaperone role on HrcQ, HrcQ_C_ likely participates as a structural component of the T3SS sorting platform.

It is now commonly admitted that T3SSs and flagellum have coevolved. Mariano et al. focus on the FliF subunit of the flagellum MS ring. They provide evidence on the oligomerization states of the FliF periplasmic Ring-Building Motifs (RBM) demonstrating that contrarily to RBM1, RBM2 and RBM3 form ring-like structures. They also report that RBM1 impacts RBM2 oligomerization and propose a role of the FliF RBMs for assembly of the MS ring and for the different symmetries adopted by these domains. Minamino et al. detail the role of chaperones required for formation of the flagellar filament. They show that three chaperones, FlgN, FliS and FliT are necessary for formation of robust filaments, as a triple mutant yields shorter filaments and releases unassembled filament subunit FliC in large amount in the medium. They then isolated suppressive mutations of the triple mutant, all located in FliC and destabilizing the FliC structure, suggesting that the role of the chaperones is to maintain FliC under an unfolded state for its efficient transport.

YadA is a trimeric autotransporter that serves as adhesin to facilitate adhesion of the bacterial cell onto the target host tissues. Using a battery of *in vivo* and *in vitro* assays, Meuskens et al. demonstrate that the *Yersinia enterocolitica* YadA adhesin does not only interact with host matrix protein such as fibronectin and collagen but also binds to glycan moieties, and notably with the N-linked glycans of the vitronectin and of heparin.

In the following review Yang et al. summarize the current knowledge and discuss the relationship between type VI secretion and metals. The T6SS, which is known as one of the key players in bacterial competition by directly injecting toxic effectors into the target bacterium, also participates to exploitative competition by collecting metals in the environment. They describe how T6SS secretes proteins that bind zinc, manganese, iron, copper or molybdenum, and what are the processes employed to take them back once loaded.

Robinson et al. performed a bioinformatic analyses of type VI secretion system (T6SS) gene clusters in *Campylobacter jejuni* strains to provide information on the prevalence of T6SS gene clusters and their genetic organization. Analyses of the *vgrG* genes and their neighborhoods allowed to list potential effectors with putative nuclease, lipase or peptidoglycan hydrolase activities. Amaya et al. provide evidence that the two T6SS associated to the SPI-6 and SPI-19 islands in the cattle-adapted *Salmonella enterica* Dublin pathogen participate to interbacterial competition. Comparative genomics allowed the authors to identify candidate effectors-immunity pairs, which were further shown to contribute to the antibacterial activity. These two works will be likely the bases for further studies aimed at better understanding the function of these T6SSs and the activities of the effectors delivered by these machines.

Fromm and Dehio review *Bartonella* T4SS effectors and their role on host cells. In addition to carrying a C-terminal bipartite Bep intracellular delivery (BID) domain responsible for selection and transport by the T4SS, most *Bartonella* effectors possess a FIC domain that mediate AMPylation of target proteins in the host. In addition, some T4SS effectors contains phosphorylation motifs that facilitate interaction with host proteins.

Further information on conjugative T4SSs is provided by the next two articles. Carranza et al. engineered functional fluorescent reporter fusions to the TrwB coupling protein and TrwK VirB4-like protein, which both associate to the inner membrane portion of the R388 conjugative T4SS, as well as to the TrwC relaxase. Fluorescence microscopy and super-resolution dSTORM revealed that while TrwC is diffuse in the cytoplasm, TrwB and TrwK localize as patches in the membrane. Interestingly they observed that TrwK segregated in a very few loci per cell, likely marking T4SSs, whereas the number of TrwB foci was much more important, suggesting that TrwB is more labile and can associate/dissociate from the T4SS. Interestingly, the three proteins significantly recoloalize to form polar foci during mating, at the sites of contact with the recipient cell. Callaghan et al. provide information on the ParA and ParB partitioning proteins associated to the T4SS in *Neisseria gonorrhoeae*. They present evidence that ParA and ParB translation is under the control of a riboswitch and that a stem loop in the 5′-UTR region participate in *parAB* regulation. Then then show that both ParA and ParB interact with the TraI relaxase, supporting the hypothesis that the ParAB complex facilitates TraI-mediated nicking at the site of secretion.

Finally, Grossman et al. present new information on the recently identified T11SS. The T11SS is comprised of an outer membrane β-barrel. In *Xenorhabdus nematophila*, the gene encoding the NilB T11SS barrel is coregulated with *nilC*, encoding a lipoprotein. Here, they show that NilC is surface-exposed in both *E. coli* and *X. nematophila* in a NilB-dependent manner, in agreement with the observation that NilC carries a T11SS-targeting C-terminal 8-stranded β-barrel.

Taken together, the reviews and articles published in this issue cover many aspects of macromolecule export and secretion in bacteria, from the prevalence and genetic organization of the gene clusters encoding these machines to mechanistic insights on how effectors are recruited and transported. It also highlights that this topic of research is very active and that many exciting discoveries are ahead of us.

## Author Contributions

IA and EC were the editors of the Research Topic “*Bacterial Secretion Systems, Volume II*.” Both authors contributed to the article and approved the submitted version.

## Conflict of Interest

The authors declare that the research was conducted in the absence of any commercial or financial relationships that could be construed as a potential conflict of interest.

## Publisher's Note

All claims expressed in this article are solely those of the authors and do not necessarily represent those of their affiliated organizations, or those of the publisher, the editors and the reviewers. Any product that may be evaluated in this article, or claim that may be made by its manufacturer, is not guaranteed or endorsed by the publisher.

